# Supplementary material for: Heat adaptation of phage T7 under an extended genetic code
Source: Virus Evol. 2021 Dec 1;7(2):veab100. doi: 10.1093/ve/veab100 (PMC8923235; doi:10.1093/ve/veab100)
Supplement: veab100_Supp [file veab100_supp.zip › Sequencing_Analysis.zip!/Sequencing_Analysis/A3/evidence/SNP_9.html]

BRESEQ :: Evidence


| Predicted mutation | | | | | | |
| --- | --- | --- | --- | --- | --- | --- |
| evidence | seq id | position | mutation | annotation | gene | description |
| RA | NC\_001604.1 | 26,746 | G→T | intergenic (–/–) | – / – | –/– |

| Read alignment evidence... | | | | | | | | | | | |
| --- | --- | --- | --- | --- | --- | --- | --- | --- | --- | --- | --- |
|  | seq id | position | | ref | new | freq | score (cons/poly) | reads | annotation | genes | product |
| \* | NC\_001604.1 | 26,746 | 0 | G | T | 99.1% | 3512.6 / 1.0 | 1588 | intergenic (–/–) | *–/–* | –/– |
| Reads supporting (aligned to +/- strand):  **ref** base G (4/10);  **new** base T (763/806);  **total** (769/819) | | | | | | | | | | | |
| Fisher's exact test for biased strand distribution *p*-value = 1.81e-01 | | | | | | | | | | | |
| Kolmogorov-Smirnov test that lower quality scores support variant *p*-value = 9.99e-01 | | | | | | | | | | | |

Only 100 of 1588 total aligned reads displayed.  

`GGCAACAGTCGTGGTCTCATTGGGACTTTGGGGAAAACGTACAGGTTCTAGCTTGTCAGAGTATCAGCTCAGATATGTATGTGATTCTTCGCAATGAGTTCAATACGTTCCTAGCTAGAATCTCTTTCACTAAGAACGCCATTGACTTACAGGGAGAACCCTATCGTGCCTTTATGGACATGAAGATTCGATACACGATTCCTAGTGGAACATACAACGATGACACATTCACTACCTCTATTCATATTCCAACAATTTATGGTGCAAACTTCGGGAGGGGCAAAATCACTGTATTGGAGCCTGATGGTAAGATAACCGTGTTTGAGCAACCTACGGCTGGGTGGAATAGCGACCCTTGGCTGAGACTCAGCGGTAACTTGGAGGGACGCATGGTGTACATTGGGTTCAACATTAACTTCGTATATGAGTTCTCTAAGTTCCTCATCAAGCAGACTGCCGACGACGGGTCTACCTCCACGGAAGACATTGGGCGCTTACAGTTACGCCGAGCGTGGGTTAACTACGAGAACTCTGGTACGTTTGACATTTATGTTGAGAACCAATCGTCTAACTGGAAGTACACAATGGCTGGTGCC  >  NC_001604.1/26448‑27043`  

                                                                                                                                                                                                                                                                                                          `|`                                                                                                                                                                                                                                                                                                           
`tataagagacagggTCTCATTGGGACTTTGGGGAAAACGTACAGGTTCTAGCTTGTCAGAGTATCAGCTCAGATATGTATGTGATTCTTCGCAATGAGTTCAATACGTTCCTAGCTAGAATCTCTTTCACTAAGAACGCCATTGACTTACAGGGAGAACCCTATCGTGCCTTTATGGACATGAAGATTCGATACACGATTCCTAGTGGAACATACAACGATGACACATTCACTACCTCTATTCATATTCCAACAATTTATGGTGCAAACTTCGGGAGGGGCAAAATCACTGTATTGGATcc                                                                                                                                                                                                                                                                                                         <  1:85170/289‑1 (MQ=255)`  
`atgtgtataagagacagcATTGGGACTTTGGGGAAAACGGACAGGTTCTAGCTTTTCAGAGTATCAGCTCAGATATGTATGTGATTCTTCGCAATGAGTTCAATACGTTCCTAGCTAGAATCTCTTTCACTAAGAACGCCATTGACTTACAGGGAGAACCCTATCGTGCCTTTATGGACATGAAGATTCGATACACGATTCCTAGTGGAACATACAACGATGACACATTCACTACCTCTATTCATATTCCAACAATTTATGGTGCAAACTTCGGGAGGGGCAAAATCACTGTATTGGATcc                                                                                                                                                                                                                                                                                                         <  1:326797/284‑1 (MQ=255)`  
       `gTCGTGGTCTCATTGGGACTTTGGGGAAAACGTACAGGTTCTAGCTTGTCAGAGTATCAGCTCAGATATGTATGTGATTCTTCGCAATGAGTTCAATACGTTCCTAGCTAGAATCTCTTTCACTAAGAATGCCATTGACTTACAGGGAGAACCCTATCGTGCCTTTATGGACCTGAAGATTCGATACACGATTCCTAGTGTAACATACAACGATGCCACATTCCCTACCTCTATTCATATTCCAACACTTAATGGTGCAAACTTCGGTATGGGCAAAATCACTGTATTGGATCCTGATGGt                                                                                                                                                                                                                                                                                                  >  1:147415/1‑301 (MQ=255)`  
       `gTCGTGGTCTCATTGGGACTTTGGGGAAAACGTACAGGTTCTAGCTTGTCAGAGTATCAGCTCAGATATGTATGTGATTCTTCGCAATGAGTTCAATACGTTCCTAGCTAGAATCTCTTTCACTAAGAACGCCATTGACTTACAGGGAGAACCCTATCGTGCCTTTATGGACATGAAGATTCGATACACGATTCCTAGTGTAACATACAACGATGACACATTCACTACCTCTATTCATATTCCAACAATTTATGGTGCAAACTTCGGGAGGGGCAAAATCACTGTATTGGATCCTGATGGt                                                                                                                                                                                                                                                                                                  >  1:248238/1‑301 (MQ=255)`  
       `gTCGTGGTCTCATTGGGAATTTGGGGAAAACGTACAGGTTCTAGCTTGTCAGAGTATCAGCTCAGATATGTATGTGATTCTTCGCAATGAGTTCAATACGTTCCTAGCTAGAATCTCTTTCACTAAGAACGCCATTGACTTACAGGGAGAACCCTATCGTGCCTTTATGGACATGAAGATTCGATAAACGATTCCTAGTGCAACATACAACGATGACACATTCACTACCTCTATTCATATTCCAACAATTTATGGTGCAAACTTCGGGAGGGGCAAAATCACTGTATTGGATCCTGATGGt                                                                                                                                                                                                                                                                                                  >  1:307151/1‑301 (MQ=255)`  
         `cGTGGTCTCATTGGGACTTTGGGGAAAACGTACAGGTTCTAGCTTGTCAGAGTATCAGCTCAGATATGTATGTGATTCTTCGCAATGAGTTCAATACGTTCCTAGCTAGAATCTCTTTCACTAAGAACGCCATTGACTTACAGGGAGAACCCTATCGTGCCTTTATGGACATGAAGATTCGATACACGATTCCTAGTGGAACATACAACGATGACACATTCACTACCTCTATTCATATTCCAACAATTTATGGTGCAAACTTCGGGAGGGGCAAAATCACTGTATTGGATCCTGATGGTaa                                                                                                                                                                                                                                                                                                >  1:241895/1‑301 (MQ=255)`  
               `ctcATTTGGACTTTGGGGAAAACGTACAGGTTCTAGCTTGTCAGAGTATCAGCTCAGATATGTATGTGATTCTTCGCAATGAGTTCAATACTTTCCTAGCTAGAATCTCTTTCACTAAGAACGCCATTTACTTACAGTGAGAACCCTATCGTGCCTTTATGGACATGAAGATTCGATACACGATTCCTAGTTGAACATACAACGATGACACATTCACTACCTCTATTCCTATTCCAACAATTTATGGTGCAAACTTCGGGAGGGGCCAAATCACTGTATTGGATCCTGATGGTACGTTAAc                                                                                                                                                                                                                                                                                          >  1:65473/1‑301 (MQ=255)`  
                 `cATTGGGACTTTGGGGAAAACGTACAGGTTCTAGCTTGTCAGAGTATCAGCTCAGATATGTATGTGATTCTTCGCAATGAGTTCAATACGTTCCTAGCTAGAATCCCTTTCACTAAGAACGCCATTGACTTACAGGGAGAACCCTATCGTGCCTTTATGGACATGAAGATTCGATACACGATTCCTAGTGGAACATACAACGATGACACATTCACTACCTCTATTCATATTCCAACAATTTATGGTGCAAACTTCGGGAGGGGCAAAATCACTGTATTGGATCCTGATGGTactgtctctt                                                                                                                                                                                                                                                                                        >  1:103081/1‑292 (MQ=255)`  
                 `cATTGGGACTTTGGGGAAAACGTACAGGTTCTAGCTTGTCAGAGTATCAGCTCAGATATGTATGTGATTATTCGCAATGAGTTCAATACGTTCCTAGCTAGAATCTCTTTCACTAAGAACGCCATTGACTTACAGGGAGAACCCTATCGTGCCTTTATGGACATGAAGATTCGATACACGATTCCTAGTGGAACATACAACGATGACACATTCACTACCTCTATTCATATTCCAACAATTTATGGTGCAAACTTCGGGAGGGGCAAAATCACTGTATTGGATCCTGATGGTAAGATAACCg                                                                                                                                                                                                                                                                                        >  1:317807/1‑301 (MQ=255)`  
                         `ttttAGGGAAAACGTACAGATTCTAGCTTGTCAGTGGATCAGCTCAGATATGTATGTGATTCTTCGAAATGAGTTCAATACGTTCCTAGCTAGAATCTCTTTCACTAAGAACGCCATTGACTTACAGGGAGAACCCTATCGTGCCTTTATGGACATGAAGATTCGATACACGATTCCTAGTGGAACATACAACGATGACACATTCACTACCTCTATTCATATTCCAACAATTTATGGTGCAAACTTCGGGAGGGGCAAAATCACTGTATTGGATCCTGATGGTAAGATAACCGTGTTTGAg                                                                                                                                                                                                                                                                                <  1:313643/300‑1 (MQ=255)`  
                              `gggAAAACGTACAGGTTATAGCTTGTCAGAGTATCAGCTCAGATATGTATGTGATTCTTCGCAATGAGTTCAATACGTTCCTAGCTAGAATCTCTTTCACTAAGAACGCCATTGACTTACAGGGAGAACCCTATCGTGCCTTTATGGACATGAAGATTCGATACACGATTCCTAGTGGAACATACAACGATGACACATTCACTACCTCTATTCATATTCCAACAAGTTATGGTGCAAACTTCGGGAGGGGCAAAATCACTGTATTGGATACTGATGGTAAGATAACCGTGTTTGAGCAAcc                                                                                                                                                                                                                                                                           <  1:187155/301‑1 (MQ=255)`  
                                    `acttACAGGTTCTATCTTGTCAGAGTATCAGCTCAGATATGTATGTGATTCTTCGCAATGAGTTCAATACGTTCCTAGCTAGAATCTCTTTCACTAAGAACGCCATTGACTTACAGGGAGAACCCTATCGTGCCTTTATGGACATGAAGATTCGATACACGATTCCTAGTGGAACATACAACGATGACACATTCACTACCTCTATTCATATTCCAACAATTTATGGTGCAAACTTCGGGAGGGGCAAAATCACTGTATTGGATCCTGATGGTAAGATAACCGTGTTTGAGCAACCTACGGc                                                                                                                                                                                                                                                                     <  1:223830/298‑1 (MQ=255)`  
                                     `agacagggTTCTAGCTTGTCAGAGTATCAGCTCAGATATGTATGTGATTCTTCGCAATGAGTTCAATACGTTCCTAGCTAGAATCTCTTTCACTAAGAACGCCATTGACTTACAGGGAGAACCCTATCGTGCCTTTATGGACATGAAGATTCGATACACGATTCCTAGTGGAACATACAACGATGACACATTCACTACCTCTATTCATATTCCAACAATTTATGGTGCAAACTTCGGGAGGGGCAAAATCACTGTATTGGATCCTGATGGTAAGATAACCGTGTTTGAGCAACCTACGGCt                                                                                                                                                                                                                                                                    <  1:2337/295‑1 (MQ=255)`  
                                           `ggTTCTAGCTTGTCAGAGTATCAGCTCAGATATGTATGTGATTCTTCGCAATGAGTTCAATACGTTTCTAGCTAGAATCTCTTTCACTAAGAACGCCATTGACTTACAGGGAGAACCCTATCGTGCCGTTCTGGACATGCAGATTCGATACACGATTCCTAGTGGAACATACAACGATGACACATTCACTACCTCTATTCATATTCCAACAATTTATGGTGCAAACTTCGGGAGCGGCATAATCACTGTATTGGATCCTGATGGTAAGATAACCGTGTTTGAGCACCCTACGGCTGGGttg                                                                                                                                                                                                                                                              >  1:269721/1‑299 (MQ=255)`  
                                           `ggTTCTAGCTTGTCAGAGTATCAGCTCAGATATGTATGTGATTCTTCGCAATGAGTTCAATACGTTCCTAGCTAGAATCTCTTTCACTAAGAACGCCATTGACTTACAGGGAGAACCCTATCGTGCCTTTATGGACATGAAGATTCGATACACGATTCCTAGTGGAACATACAACGATGACACATTCACTACCTCTATTCATATTCCAACAATTTATGGTGCAAACTTCGGGAGGGGCAAAATCACTGTATTGGATCCTGATGGTAAGATAACCGTGTTTGAGCAACCTACGGCTGGGttt                                                                                                                                                                                                                                                              >  1:287607/1‑299 (MQ=255)`  
                                                 `gagacagcAGAGTATCAGCTCAGATATGTATGTGATTCTTCGCAATGAGTTCAATACGTTCCTAGCTAGAATCTCTTTCACTAAGAACGCCATTGACTTACAGGGAGAACCCTATCGTGCCTTTATGGACATGAAGATTCGATACACGATTCCTAGTGGAACATACAACGATGACACATTCACTACCTCTATTCATATTCCAACAATTTATGGTGCAAACTTCGGGAGGGGCAAAATCACTGTATTGGATCCTGATGGTAAGATAACCGTGTTTGAGCAACCTACGGCTGGGTGGAATAGc                                                                                                                                                                                                                                                        <  1:332910/294‑1 (MQ=255)`  
                                                   `agagacaaagTATCAGCTCAGATATGTTTGTGATTCTTCGCAATGAGTTCAATACGTTCCTAGCTAGAATCTCTTTCACTAAGAACGCCATTGACTTACAGGGAGAACCCCATCGTGCCTTTATGGACATGAAGATTCGATACACGATTCCTAGTGGAACATACAACGATGACACATTCACTACCTCTATTCATATTCCAACAATTTATGGTGCAAACTTCGGGAGGGGCAAAATCACTGTATTGGATCCTGATGGTAAGATAACCGTGTTTGAGCAACCTACGGCTGGGTGGAATAGCGa                                                                                                                                                                                                                                                      <  1:244674/293‑1 (MQ=255)`  
                                                      `ttCAGAGTATCAGCTCAGATATGTATGTGATTCTTCGCAATGAGTTCAATACGTTCCTAGCTAGAATCTCTTTCACTAAGAACGCCATTGACTTACAGGGAGAACCCTATCGTGCCTTGATGGACATGAAGATTCGATACACGATTCCTAGTGGAACATACAACGATGACACATTCACTACCTCTATTCATATTCCAACAATTTATGGTGCAAACTTCGGGAGGGGCAAAATCACTGTATTGGATCCTGATGGTAAGATAACCGTGTTTGAGCAACCTACGGCTGGGTGGAATAGCGAccc                                                                                                                                                                                                                                                   <  1:190276/300‑1 (MQ=255)`  
                                                            `gTATCAGCTCAGATATGTATGTGATTCTTCGCAATGAGTTCAATACGTTCCTAGCTAGAATCTCTTTCACTAAGAACGCCATTGACTTACAGGGAGAACCCTATCGTGCCTTTATGGACATGAAGATTCGATACACGATTCCTAGTGGAACATACAACGATGACACATTCACTACCTCTATTCATATTCCAACAATTTATGGTTCAAACTTCGGGAGGGGCAAAATCACTGTATTGGATCCTGATGGTAAGATAACCGTGTTTGAGCAACCTACGGCTGGGTGGAATAGCGACCCTTGGCt                                                                                                                                                                                                                                             >  1:154682/1‑301 (MQ=255)`  
                                                                 `gtataagagacagatgtGATTCTTCGCAATGAGTTCAATACGTTCCTAGCTAGAATCTCTTTCACTAAGAACGCCATTGACTTACAGGGAGAACCCTATCGTGCCTTTATGGACATGAAGATTCGATACACGATTCCTAGTGGAACATACAACGATGACACATTCACTACCTCTATTCATATTCCAACAATTTATGGTGCAAACTTCGGGAGGGGCAAAATCACTGTATTGGATCCTGATGGTAAGATAACCGTGTTTGAGCAACCTACGGCTGGGTGGAATAGCGACCCTTGGCTGAGAc                                                                                                                                                                                                                                        <  1:300970/288‑1 (MQ=255)`  
                                                                 `aGCTCAGATATGTATGTGATTCTTCGCAATGAGTTCAATACGTTCCTAGCTAGAATCTCTTTCACTAAGAACGCCATTGACTTACAGGGAGAACCCTATCGTGCCTTTATGGACATGAAGATTCGATACACGATTCCTAGTGGAACATACAACGATGACACATTCACTACCTCTATTCATATTCCAACAATTTATGGTGCAAACTTCGGGAGGGGCAAAATCACTGTATTGGATCCTGATGGTAAGATAACCGTGTTTGAGCAACCTACGGCTGGGTGGAATAGCGACCCTTGGCTGAGAc                                                                                                                                                                                                                                        <  1:323194/301‑1 (MQ=255)`  
                                                                 `aGCTCAGATATGTATGTGATTCTTCGCAATGAGTTCAATACGTTCCTAGCTAGAATCTCTTTCACTAAGAACGCCATTGACTTACAGGGAGAACCCTATCGTGCCTTTATGGACATGAAGATTCGATACACGATTCCTAGTGGAACATACAACGATGACACATTCACTACCTCTATTCATATTCCAACAATTTATGGTGCAAACTTCGGGAGGGGCAAAATCACTGTATTGGATCCTGATGGTAAGATAACCGTGTTTGAGCAACCTACGGCTGGGTGGAATAGCGACCCTTGGCTGAGAc                                                                                                                                                                                                                                        <  1:265797/301‑1 (MQ=255)`  
                                                                  `gCTCAGATATGTATGTGATTCTTCGCAATGAGTTCAATACGTTCCTAGCTAGAATCTCTTTCACTAAGAACGCCATTGACTTACAGGGAGAACCCTATCGTGCCTTTATGGACATGAAGATTCGATACACGATTCCTAGTGGAACATACAACGATGACACATTCACTACCTATATTCATATTCCAACAATTTATGGTGCAAACTTCGGAAGGGGCAAAATCACTGTATTGGATCCTGATGGTAAGATAACCGTGTTTGAGCAACCTACGCCTGGGTGGAATAGCGACCCTTGGCTGCGACt                                                                                                                                                                                                                                       >  1:132196/1‑301 (MQ=255)`  
                                                                       `gATATGTATGTGATTCTTCGCAATGAGTTCAATACGTTCCTAGCTAGAATCTCTTTCACTAAGAACGCCATTGACTTACAGGGAGAACCCTATCGTGCCTTTATGGACATGAAGATTCGATACACGATTCCTAGTGGAACATACAACGATGACACATTCACTACCTCTATTCATATTCCAACAATTTATGGTGCAAACTTCGTGAGGGGCAAAATCACTGTATTGGATCCTGATGGTAAGATAACCGTGTTTGAGCAACCTACGGCTGGGTGGAATAGCGACCCTTGGCTGAGACTCAGCg                                                                                                                                                                                                                                  >  1:340910/1‑301 (MQ=255)`  
                                                                            `gtatgtGATTCTTCGCAATGAGTTCAATACGTTCCTAGCTAGAATCTCTTTCACTAAGAACGCCATTGACTTACAGGGAGAACCCTATCGTGCCTTTATGGACATGAAGATTCGATACACGATTCCTAGTGGAACATACAACGATGACACATTCACTACCTCTATTCATATTCCAACAATTTATGGTGCAAACTTCGGGAGGGGCAAAATCACTGTATTGGATCCTGATGGTAAGATAACCGTGTTTGAGCAACCTACGGCTGGGTGGAATAGCGACCCTTGGCTGAGACTCATCGGTAAc                                                                                                                                                                                                                             >  1:183785/1‑301 (MQ=255)`  
                                                                             `tatgtGATTCTTCGCAATGAGTTCAATACGTTCCTAGCTAGAATCTCTTTCACTAAGAACGCCATTGACTTACAGGGAGAACCCTATCGTGCCTTTATGGACATGAAGATTCGATACACGATTCCTCGTGGAACATACAACGATGACACATTCACTCCCTCTCTTCATATTCCAACAATTTATGGTGCAAACTTCGGGAGGGGCAAAATCCCTGTATTGGATCCTGATGGTAAGATAACCGTGTTTGAGCAACCTACGGCTGGGTGGAATAGCGAcctgtctcttctacacatctccgagc                                                                                                                                                                                                                            >  1:181318/1‑277 (MQ=255)`  
                                                                             `aagagacagcttcGCAATGAGTTCACGACGTTACTAGCTAGAATCTATTTCACTAAGAACGCCATTAACTTACAGAGAGAACCCTATCGTGCCTTTATGGACATGAAGATTCGATACACGATTCCTAGTGGAACATACAACGATGACACATTCACTACCTCTATTCATATTCCAACAATTTATGGTGCAAACTTCGGGAGGGGCAAAATCACTGTATTGGATCCGGATGGTAAGATAACCGTGTTTGAGCAACCTACGGCTGGGTGGAATAGCGACCCTTGGCTGAGACTCAGCGGTAACt                                                                                                                                                                                                                            <  1:172203/292‑1 (MQ=255)`  
                                                                              `atgtGATTCTTCGCAATGAGTTCAATACGTTCCTAGCTAGAATCTCTTTCACTAAGAACGCCATTGACTTACAGGGAGAACCCTATCGTGCCTTTATGGACATGAAGATTCGATACACGATTCCTAGTGGAACATACAACGATGACACATTCACTACCTCTATTCATATTCCAACAATTTATGGTGCAAACTTCGGGAGGGGCAAAATAACTGTATTGGATCCTGATGTTAATATAACCGTGTTTGAGCAACCTACGGCTGGGTGGAATAGCAACACTTGGCGGAGACTCAGTGGTAACtt                                                                                                                                                                                                                           >  1:50089/1‑301 (MQ=255)`  
                                                                              `atgtGATTCTTCGCAATGAGTTCAATACGTTCCTAGCTAGAATCTCTTTCACTAAGAACGCCATTGACTTACAGGGAGAACCCTATCGTGCCTTTATGGACATGAAGATTCGATACACGATTCCTAGTGGAACATACAACGATGACACATTCACTACCTCTATTCATATTCCAACAATTTATGGTGAAAACTTCGGGAGGGGCAAAATCACTGTATTTGATCCTGATGGTAAGATAACCGTGTTTGAGCAACCTACGGCTGGGTGGAATAGCTACCCTTGGCTGAGACTCAGCGGTAACtt                                                                                                                                                                                                                           >  1:272697/1‑301 (MQ=255)`  
                                                                                        `tcGCAATGAGTTCAATACGTTCCTAGCTAGAATCTCTTTCACTAAGAACGCCATTGACTTACAGGGAGAACCCTATCGTGCCTTTATGGACATGAAGATTCGATACACGATTCCTAGTGTAACATACAACGATGACACATTCACTACCTCTATTCATATTCCAACAATTTATGGTGCAAACTTCGGGAGGGGCAAAATCACTGTATTGGATCCTTATGGTAAGATAACCGTGTTTGAGCAACCTACGGCTGGGTTGAATAGCGACCCTTGGCTGAGACTCAGCGGTTACTTGGAGGGActc                                                                                                                                                                                                                 >  1:87379/1‑299 (MQ=255)`  
                                                                                           `cAATGAGTTCAATACGTTCCTAGCTAGAATCTCTTTCACTAAGAACGCCATTGACTTACAGGGAGAACCCTATCGTGCCTTTATGGACATGAAGATTCGATACACGATTCCTAGTGGAACATACAACGATGACACATTCACTACCTCTATTCATATTCCAACAATTTATGGTGCAAACTTCGGGAGGGGCAAAATCACTGTATTGGATCCTGATGGTAAGATAACCGTGTTTGAGCAACCTAAGGACGGGGGGAAGAGCGACAATTAGCTGAGACTCAGAGATAAGTTGGAGGGTGGCATg                                                                                                                                                                                                              >  1:216752/1‑301 (MQ=255)`  
                                                                                               `gAGTTCAATACGTTCCTAGCTAGAATCTCTTTCACTAAGAACGCCATTGACTTACAGGGAGAACCCTATCGTGCCTTTATGGACATGAAGATTCGATACACGATTCCTAGTGGAACATACAACGATGACACATTCACTACCTCTATTCATATTCCAACAATTTATGGTGCAAACTTCGGGAGGGGCAAAATCACTGTATTGGATCCTGATGGTAAGATAACCGTGTTTGAGCAACCTACGGCTGGGTGGAATAGCGACCCTTGGCTGAGACTCAGCGGTAACTTGGGGGGACGCATGgggt                                                                                                                                                                                                          >  1:263047/1‑301 (MQ=255)`  
                                                                                               `gAGTTCAATACGTTCCTAGCTAGAATCTCTTTCACTAAGAACGCCATTGACTTACAGGGAGAACCCTATCGTGCCTTTATGGACATGAAGATTCGATACACGATTCCTAGTGGAACATACAACGATGACACATTCACTACCTCTATTCATATTCCAACAATTTATGGTGCAAACTTCGGGAGGGGCAAAATCACTGTATTGGATCCTGATGGTAAGATAACCGTGTTTGAGCAACCTACGGCTGGGTGGAATAGCGACCCTTGGCTGAGACTCAGCGGTAACTTGGAgggctgtctcttat                                                                                                                                                                                                          >  1:94752/1‑290 (MQ=255)`  
                                                                                                 `gTTCAATACGTTCCTAGCTAGAATCTCTTTCACTAAGAACGCCATTGACTTACAGGGAGAACCCTATCGTGCCTTTATGGACATGAAGATTCGATACACGATTCCTAGTGGAACATACAACGATGACACATTCACTACCTCTATTCATATTCCAACAATTTATGGTGCAAACTTCGGGAGGGGCAAAATCACTGTATTGGATCCTGATGGTAAGATAACCGTGTTTGAGCAACCTACGGCTGGGTGGAATAGCGACCCTTGGCTGAGACTCAGCGGTAACTTGGAGGGACGCATGGTGTAc                                                                                                                                                                                                        >  1:4022/1‑301 (MQ=255)`  
                                                                                                 `gTTCAATACGTTCCTAGCTAGAATCTCTTTCACTAAGAACGCCATTGACTTACAGGGAGAACCCTATCGTGCCTTTATGGACATGAAGATTCGATACACGATTCCTAGTGGAACATACAACGATGACACATTCACTACCTCTATTCATATTCCAACAATTTATGGTGCAAACTTCGGGAGGGGCAAAATCACTGTATTGGATCCTGATGGTAAGATAACCGTGTTTGAGCAACCTACGGCTGGGTGGAATAGCGACCCTTGGCTGAGACTCAGCGGTAACTTGGAGGGACGCATGGTGTAc                                                                                                                                                                                                        <  1:97249/301‑1 (MQ=255)`  
                                                                                                 `gTTCAATACGTTCAGAGCTAGAATCTCTTTCACTAAGAACGCCATTGACTTACAGGGAGAACCCTATCGTGCCTTTATGGACATGAAGATTCGATACACGATGCCTAGTGGAACATACAACGATGACACATTCACTACCTCTATTCATATTCCAACAATTTATGGTGCAAACTTCGGGAGGGGCAAAATCACTGTATTGGATCCTGATGGTAAGATAACCGTGTTTGAGCAACCTACGGCTGGGTGGAATAGCGACCCTTGGCTGAGACTCAGCGGTAACTTGGAGGGACGCATGGTGTAc                                                                                                                                                                                                        <  1:54001/301‑1 (MQ=255)`  
                                                                                                   `tCAATACGTTCCTAGCTAGAATCTCTTTCACTAAGAACGCCATTGACTTACAGGGAGAACCCTATCGTGCCTTTATGGACATGAAGATTCGATACACGATTCCTAGTGGAACATACAACGATGACACATTCACTACCTCTATTCATATTCCAACAATTTATGGTGCAAACTTCGGGAGGGGCAAAATCACTGTATTGGATCCTGATGGTAAGATAACCGTGTTTGAGCAACCTACGGCTGGGTGGAATAGCGACCCTTGGCTGAGACTCAGCGGTAACTTGGAGGGACGCATGGTGTACAt                                                                                                                                                                                                      >  1:56750/1‑301 (MQ=255)`  
                                                                                                    `aaaTACGTTCCTAGCTAGAAACTATTTCACTAAGAACGCCATTGACTTACAGGGAGAACCATATCGTGCCTTTATGGACATGAAGATTCGATACACGATTCCTAGTGGAACATACAACGATGACACATTCACTACCTCTATTCATATTCCAACAATTTATGGTGCAAACTACGGGAGGGGCAAAATCACTGTATTGGATCCTGATGGTAAGATAACCGTGTTTGAGCAACCTACGGCTGGGTGGAATAGCGACCCTTGGCTGAGACTCAGCGGTAACTTGGAGGGACGCATGGTGTACAtt                                                                                                                                                                                                     <  1:201438/300‑1 (MQ=255)`  
                                                                                                        `aCGTTCCTAGCTAGAATCTCTTTCACTAAGAACGCCATTGACTTACAGGGAGAACCCTATCGTGCCTTTATGGACATGAAGATTCGATACACGATTCCTAGTGGAACATACAACGATGACACATTCACTACCTCTATTCATATTCCAACAATTTATGGTGCAAACTTCGGGAGGGGCAAAATCACTGTATTGGATCCTGATGGTAAGATAACCGTGTTTGAGCAACCTACGGCTGGGTGGAATAGCGACCCTTGGCTGAGACTCAGCGGTAACTTGGAGGGACGCATGGTGTACATTGGGt                                                                                                                                                                                                 <  1:113171/301‑1 (MQ=255)`  
                                                                                                          `gTTCCTAGCTAGAATCTCTTTCACTAAGAACGCCATTGACTTACAGGGAGAACCATATCGTGCCTTTATGGACATGAAGATTCGATACACGATTCCTAGTGGAACATACAACGATGACACATTCACTACCTCTATTCATATTCCAACAATTTATGGTGCAAACTTCGGGAGGGGCAAAATCACTGTATTGGATCCTGATGGTAAGATAACCGTGTTTGAGCAACCTACGGCAGGGTGGAATAGCGACCCTTGGCTGAGACTCAGCGGTAAATAGGAGGGACGCATGGGGTACATTGGGTTc                                                                                                                                                                                               >  1:281032/1‑301 (MQ=255)`  
                                                                                                               `tagctagAATCTCTTTCACTAAGAACGCCATTGACTTACAGGGAGAACCCTATCGTGCCTTTATGGACATGAAGATTCGATACACGATTCCTAGTGGAACATACAACGATGACACATTCACTACCTCTATTCATATTCCAACAATTTATGGTGCAAACTTCGGGAGGGGCAAAATCACTGTATTGGATCCTGATGGTAAGATAACCGTGTTTGAGCAACCTACGGCTGGGTGGAATAGCGACCCTTGGCTGAGACTCAGCGGTAACTTGGAGGGACGCATGGTGTACATTGGGTTCAACAt                                                                                                                                                                                          <  1:161069/301‑1 (MQ=255)`  
                                                                                                                    `agAATCTCTTTCACTAAGAACGCCATTGACTTACAGGGAGAACCCTATCGTGCCTTTATGGACATGAAGATTCGATACACGATTCCTAGTGGAACATACAACGATGACACATTCACTACCTCTATTCATATTCCAACAATTTATGGTGCAAACTTCGGGAGGGGCAAAATCACTGTATTGGATCCTGATGGTAAGATAACCGTGTTAGAGCAACCTACGGCTGGGTGGAATAGCGACCCTTGGCTGAGACTCAGCGGTAACTTGGAGGGACGCATGGTGTACATTGGGTTCAACATTAACt                                                                                                                                                                                     <  1:166679/301‑1 (MQ=255)`  
                                                                                                                     `aaagctctTTCACTAAGAACGCCATTGACTTACAGGGAGAACCCTATCGTGCCTTTATGGACATGAAGATTCGATACACGATTCCTAGTGGAACATACAACGATGACACATTCACTACCTCTATTCATATTCCAACAATTTATGGTGCAAACTTCGGGAGGGGCAAAATCACTGTATTGGATCCTGATGGTAAGATAACCGTGTTTGAGCAACCTACGGCTGGGTGGAATAGCGACCCTTGGCTGAGACTCAGCGGTAACTTGGAGGGACGCATGGTGTACATTGGGTTCAACATTAACtt                                                                                                                                                                                    <  1:285515/297‑1 (MQ=255)`  
                                                                                                                       `aTCTCTTTCACTAAGAACGCCATTGACTTACAGGGAGAACCCTATCGTTCCTTTATGGACATGAAGATTCGATACACGATTCCTAGTGGAACATACAACGATGACACATTCACTACCTCTATTCATATTCCAACAATTTATGGTGCAAACTTCGGGAGGGGCAAAATCACTGTATTGGATCCTGATGGTAAGATAACCGTGTTTGAGCAACCTACGGCTGGGTGGAATAGCGACCCTTGGCTGAGACTCAGCGGTAACTTGGAGGGACGCATGGTGTACATTGGGTTCAACATTAACTTCg                                                                                                                                                                                  <  1:91591/301‑1 (MQ=255)`  
                                                                                                                            `tttCACTAAGAACGCCATTGACTTACA‑GGAGAACCCTATCGTGCCTTTATGGACATGAAGATTCGATACACGATTCCTAGTGGAACATACAACGATGACACATTAACTACCTCTATTCATATTCCAACAATTTATGGTGCAAACTTCGGGAGGGGCAAAATCATTGTATTGGATCCTGATGGTAAGATAACCGTGTTTGAGCAACCTACGGCTGGGTGGAATAGCGACCCTTGGCTGAGACTCAGCGGTAACTTGGAGGGACGCATGGTGTACATTGGGTTCAACATTAACTTCGTATATg                                                                                                                                                                            <  1:144712/301‑1 (MQ=255)`  
                                                                                                                             `ttCACTAAGAACGCCATTGACTTACAGGGAGAACCCTATCGTGCCTTTATGGACATGAAGATTCGATACACGATTCCTAGTGGAACATACAACGATGACACATTCACTACCTCTATTCATATTCCAACAATTTATGGTGCAAACTTCTGGAGGGGCAAAATCACTGTATTGGATCCTGATGGTAAGATAACCGTGTTTGAGCAACCTACTGCTGGGTGGAATAGCGACCCTTTGCTGAGACTCAGCGGTAACTTGGAGGGACGCATGGTGTACATTGGGTTCAACATTAACTTCGTATATg                                                                                                                                                                            >  1:135184/1‑301 (MQ=255)`  
                                                                                                                               `cACTAAGAACGCCATTGACTTACAGGGAGAACCCTATCGTGCCTTTATTGACATGAAGATTCGATACACGATTCCTAGTGGAACATACAACGATGACACATTCACTACCTCTATTCATATTCCAACAATTTATGGTGCAAACTTCGGGAGGGGCAAAATCACTGTATTGGATCCTGATGGTAAGATAACCGTGTTTGAGCAACCTACGGCTGGGAGGAATAGCGACCCTTGGCTGAGACTCAGCGGTAACTTGGAGGGACGCATGGTGTACATTGGGTTCAACATTAACTTCGTATATGAg                                                                                                                                                                          <  1:303778/301‑1 (MQ=255)`  
                                                                                                                                                 `cTTACAGGGAGAACCCAATCGTGCCTTTATGGACATGAAGATTCGATACACGATTCCTAGTGGAACATACAACGATGACACATTCACTACCTCTATTCATATTCCAACAATTTATGGTGCAAACTTCGGGAGGGGCAAAATCACTGTATTGGATCCTGATGGTAAGATAACCGTGTTTGAGCAACCTACGGCTGGGTGGAATAGCGACCCTTGGCTGAGACTCAGCGGTAACTTGGAGGGACGCATGGTGTACATTGGGTTCAACATTAACTTCGTATATGAGTTCTCTAAGTTCCtcatc                                                                                                                                                        <  1:20295/301‑1 (MQ=255)`  
                                                                                                                                                           `gaACCCTATCGTGCCTTTATGGACATGAAGATTCGATACACGATTCCTAGTGGAACATACAACGATGACACATTCACTACCTCTATTCATATTCCAACAATTTATGGTGCAAACTTCGGGAGGGGCAAAATCACTGTATTGGATCCTGATGGTAAGATAACCGTGTTTGAGCAACCTACGGCTGGGTGGAATAGCGACCCTTGGCTGAGACTCAGCGGTAACTTGGAGGGACGCATGGTGTACATTGGGTTCAACATTAACTTCGTATATGAGTTCTCTAAGTTCCTCATCACGCAGACtc                                                                                                                                              >  1:100012/1‑300 (MQ=255)`  
                                                                                                                                                            `aaaccTATCGTGCCTTTATGGACATGAAGATTCGATACACGATTCCCAGTGGAACATACAACGATGACACATTCACTACCTCTATTCATATTCCAACAATTTATGGTGCAAACTTCGGGAGGGGCAAAATCACTGTATTGGATACTGATGGTAAGATAACCGTGTTTGAGCAACCTACGGCTGGGTGGAATAGCGACCCTTGGCTGAGACTCAGCGGTAACTTGGAGGGACGCATGGTTTACATTGGGTTCAACATTAACTTCGTATATGAGTTCTCTAAGTTCCTCATCAAGCAGACTGc                                                                                                                                             <  1:47606/298‑1 (MQ=255)`  
                                                                                                                                                             `aCCCTATCGTGCCTTTATGGACATGAAGATTCGATACACGATTCCTAGTGGAACATACAACGATGACACATTCACTACCTCTATTCATATTCCAACAATTTATTGTGCAAACTTCTGGAGGGGCAAAATCACTGTATTGGATCCTGATGGTAAGATAACCGTGTTTGAGCAACCTACGGCTGGGTGGAATAGCGACCCTTGGCTGAGACTCAGCGGTAACTTGGAGGGACGCATGGTGTACATTGGGTTCAACATTAACTTCGTATATGAGTTCTCTAAGTTCCTCATCAAGCAGACTGcc                                                                                                                                            >  1:294831/1‑301 (MQ=255)`  
                                                                                                                                                               `ccTATCGTGCCTTTATGGACATGAAGATTCGATACACGATTCCTAGTGGAACATACAACGATGACACATTCACTACCTATATTCATATTCCAACAATTTATGGTGCAAACTTCGGGAGGGGCAAAATCACTGTATTGGATCCTGATGGTAAGATAACCGTGTTTGAGCAACCTACGGCTGGGTGGAATAGCGACCCTTGGCTGAGACTCAGCGGTAACTTGGAGGGACGCATGGTGTACATTGGGTTCAACATTAACTTCGTATATGAGTTCTCTAAGTTCCTCATCAAGCAGACTGCcga                                                                                                                                          <  1:114646/301‑1 (MQ=255)`  
                                                                                                                                                                 `tATCGTGCCTTTATGGACATGAAGATTCGATACACGATTCCTAGTGGAACATACAACGATGACACATTCACTACCTCTATTCATATTCCAACAATTTATGGTGCAAACTTCGGGAGGGGCAAAATCACTGTATTGGATCCTGATGGTAAGATAACCGTGTTTGAGCAACCTACGGCTGGGTGGAATAGCGACCCTTGGCTGAGACTCAGCGGTAACTTGGAGGGACGCATGGTGTACATTGGGTTCAACATTAACTTCGTATATGAGTTCTCTAAGTTCCTCATCAAGCAGACTGCcgacg                                                                                                                                        >  1:170428/1‑301 (MQ=255)`  
                                                                                                                                                                  `aTCGTGCCTTTATGGACATGAAGATTCGATACACGATTCCTAGTGGAACATACAACGATGACACATTCACTACCTCTATTCATATTCCAACAATTTATGGTGCAAACTTCGGGAGGGGCAAAATCACTGTATTGGATCCTGATGGTAAGATAACCGTGTTTGAGCAACCTACGGCTGGGTGGAATAGCGACCCTTGGCTGAGACTCAGCGGTAACTTGGAGGGACGCATGGTGTACATTGGGTTCAACATTAACTTCGTATATGAGTTCTCTAAGTTCCTCATCAAGCAGACTGCcgactt                                                                                                                                       >  1:278677/1‑299 (MQ=255)`  
                                                                                                                                                                  `aTCGTGCCTTTATGGACATGAAGATTCGATACACGATTCCTAGTGGAACATACAACGATGACACATTCACTACCTCTATTCATATTCCAACAATTTATGGTGCAAACTTCGGGAGGGGCAAAATCACTGTATTGGATCCTGATGGTAAGATAACCGTGTTTGAGCAACCTACGGCTGGGTGGAATAGCGACCCTTGGCTGAGACTCAGCGGTAACTTGGAAGGACGCATGGTGTACATTGGGTTCAACATTAACTTCGTATATGCGTTCTCTAAGTTCCTCATCAAGCAGACTGCcgacgc                                                                                                                                       >  1:129348/1‑300 (MQ=255)`  
                                                                                                                                                                   `gtgtataagagacagcATGAAGATTCGATACACGATTCCTAGTGGAACATACAACGATGACACATTCACTACCTCTATTCATATTCCAACAATTTATGGTGCAAACTTCGGGAGGGGCAAAATCACTGTATTGGATCCTGATGGTAAGATAACCGTGTTTGAGCAACCTACGGCTGGGTGGAATAGCGACCCTTGGCTGAGACTCAGCGGTAACTTGGAGGGACGCATGGTGTACATTGGGTTCAACATTAACTTCGTATATGAGTTCTCTAAGTTCCTCATCAAGCAGACTGCCgacgac                                                                                                                                      <  1:34929/286‑1 (MQ=255)`  
                                                                                                                                                                        `ccTTTATGGACATGAAGATTCGATACACGATTCCTAGTGGAACATACAACGATGACACATTCACTACCTCTATTCATATTCCAACAATTTATGGTGCAAACTTCGGGAGGGGCAAAATCACTGTATTGGATCCTGATGGTAAGATAACCGTGTTTGAGCAACCTACGGCTGGGTGGAATAGCGACCCTTGGCTGAGACTCAGCGGTAACTTGGAGGGACGCATGGTGTACATTGGGTTCAACATTAACTTCGTATATGAGTTCTCTAAGTTCCTCATCAAGCAGACTGCCGACGACGGGTc                                                                                                                                 >  1:195558/1‑301 (MQ=255)`  
                                                                                                                                                                        `ccTTTATGGACATGAAGATTCGATACACGATTCCTAGTGGAACATACAACGATGACACATTCACTACCTCTATTCATATTCCAACAATTTATGGTGCAAACTTCGGGAGGGGCAAAATCACTGTATTGGATCCTGATGGTAAGATAACCGTGTTTGAGCAACCTACGGCTAGGTGGAATAGCGACCCTTGGCTGAGACTCAGCGGTAACTTGGAGGGACGCATGGTGTACATTGGGTTCAACATTAACTTCGTATATGAGTTCTCTAAGTTCCTCATCAAGCAGACTGCCGACGACGGGTc                                                                                                                                 >  1:151987/1‑301 (MQ=255)`  
                                                                                                                                                                              `tGGACATGAAGATTCGATACACGATTCCTAGTGGAACATACAACGATGACACATTCACTACCTCTATTCATATTCCAACAATTTATGGTGCAAACTTCGGGAGGGGCAAAATCACTGTATTGGATCCTGATGGTAAGATAACCGTGTTTGAGCAACCTACGGCTGGGTGGAATAGCGACCCTTGGCTGAGACTCAGCGGTAACTTGGAGGGACGCATGGTGTACATTGGGTTCAACATTAACTTCGTATATGAGTTCTCTAAGTTCCTCATCAAGCAGACTGCCGACGACGGGTCTACCTc                                                                                                                           <  1:255130/301‑1 (MQ=255)`  
                                                                                                                                                                              `aggACATGAAGATTCGATACACGATTCCTAGTGGAACATACAACGATGACACATTCACTACCTCTATTCATATTCCAACAATTTATGGTGCAAACTTCGGGAGGGGCAAAATCACTGTATTGGATCCTGATGGTAAGATAACCGTGTTTGAGCAACCTACGGCTGGGTGGAATAGCGACCCTTGGCTGAGACTCAGCGGTAACTTGGAGGGACGCATGGTGTACATTGGGTTCAACATTAACTTCGTATATGAGTTCTCTAAGTTCCTCATCAAGCAGACTGCCGACGACGGGTCTACCTc                                                                                                                           <  1:62035/300‑1 (MQ=255)`  
                                                                                                                                                                                `gACATGAAGATTCGATACACGATTCCTAGTGGAACATACAACGATGACACATTCACTACCTCTATTCATATTCCAACAATTTATGGTGCAAACTTCGGGAGGGGCAAAATCACTGTATTGGATCCTGATGGTAAGATAACCGTGTTTGAGCAACCTACGGCTGGGTGGAATAGCGACCCTTGGCTGAGACTCAGCGGTAACTTGGAGGGACGCATGGTGTACATTGGGTTCAACATTAACTTCGTATATGAGTTCTCTAAGTTCCTCATCAAGAAGACTGCCGACGACGGGTCTAccctgt                                                                                                                         >  1:209042/1‑297 (MQ=255)`  
                                                                                                                                                                                              `aTACACGATTCCTAGTGGAACATACAACGATGACACATCCACTACCTCTATTCATATTCCAACAATTTATGGTGCAAACTTCGGGAGAGGCAAAATCACTGTATTGGATCCTGATGGTAAGATAACCGTGTTTGAGCAACCTACGGCTGGGTGGAATAGCGACCCTTGGCTGAGACTCAGCGGTAACTTGGAGGGACGCATGGTGTACATTGGGTTCAACATTAACTTCGTATATGAGTTCTCTAAGTTCCTCATCAAGCAGACTGCCGACGACGGGTcctgtctcttatacacatctccg                                                                                                           >  1:298027/1‑279 (MQ=255)`  
                                                                                                                                                                                                 `cacGATTCCTAGTGGAACATACAACGATGACACATTCACTACCTCTATTCATATTCCAACAATTTATGGTGCAAACTTCGGGAGGGGCAAAATCACTGTATTTGATCCTGATGGTAAGATAACCGTGTTTGAGCAACCTACGGCTGGGTGGAATAGCGACCCTTGGCTGAGACTCAGCGGTAACTTGGAGGGACTCATGGTGTACATTGGGTTCAACATTAACTTCGTATATGAGTTCTCTAAGTTCCTCATCAAGCAGACTGCCGACGACGGGTCTACCTCCACGGAAGACATTGGgcgc                                                                                                        >  1:71802/1‑301 (MQ=255)`  
                                                                                                                                                                                                          `tAGTGGAACATACAACGATGACACATTCACTACCTCTATTCATATTCCAACAATTTATGGTGCAAACTTCGGGAGGGGCAAAATCACTGTATTGGATCCTGATGGTAAGATAACCGTGTTTGAGCAACCTACGGCTGGGTGGAATAGCGACCCTTGGCTGAGACTCAGCGGTAACTTGGAGGGACGCATGGTGTACATTGGGTTCAACATTAACTTCGTATATGAGTTCTCTAAGTTCCTCATCAAGCAGACTGCCGACGACGGGTCTACCTCCACGGAAAACATTGGGCGCTTACAGttt                                                                                               >  1:38201/1‑300 (MQ=255)`  
                                                                                                                                                                                                            `gTGGAACATACAACGATGACACATTCACTACCTCTATTCATATTCCAACAATTTATGGTGCAAACTTCGGTAGGGGCAAAATCACTGTATTGGATCCTGATGGTAAGATAACCGTGTTTGAGCAACCTACGGCTGGGTGGAATAGCGACCCTTTGCTGAGACTCAGCGGTAACTTGGAGGGACGCATGGTGTACATTGGGTTCAACATTAACTTCGTATATGAGTTCTCTAAGTTCCTCATCAAGCAGACTGCCGACGACGGGTCTACCTCCACGGAAGACATTGGGCGCTTACAGTTACg                                                                                             >  1:105244/1‑301 (MQ=255)`  
                                                                                                                                                                                                            `gTGGAACATACAACGATGACACATTCACTACCTCTATTCATATTCCAACAATTTATGGTGCAAACTTCGGGAGGGGCAAAATCACTGTATTGGATCCTGATTGTAAGATAACCGTGTTTGAGCAACCTACGGCTGGGTGGAATAGCGACCCTTGGCTGAGACTCAGCGGTAACTTGGAGGGACGCATGGTGTACATTGGGTTCAACATTAACTTCGTATATGAGTTCTCTAAGTTCCTCATCAAGCAGACTGCCGACGACGGGTCTACCTCCACGGAAGACATTTGGCGCTTACAGTTAcc                                                                                             >  1:237120/1‑300 (MQ=255)`  
                                                                                                                                                                                                            `gTGGAACATACAACGATGACACATTCACTACCTCTATTCATATTCCAACAATTTATGGTGCAAACTTCGGGAGGGGCAAAATCACTGTATTGGATCCTGATGGTAACATAACCGTGTTTGAGCAACCTACGGCTGGGTGGAATAGCGACCCTTGGCTGAGACTCAGCGGTAACTTGGAGTGACGCATGGTGTACATTGGGTTCAACATTAACTTCGTATATGAGTTCTCTAAGTTCCTCATCAAGCAGACTGCCGACGACGGGTCTACCTCCACGGAAGACATTGGGCGCTTACAGTTACg                                                                                             >  1:118315/1‑301 (MQ=255)`  
                                                                                                                                                                                                            `gTGGAACATACAACGATGACACATACACTACCTCTATTCATATTCCAACAATTTATGGTGCAAACTTCGGGAAGGGCAAAATAACTGTAGTGGATCCTGATGGGAAGATAACCGTGTTTGAGCAACCTACGGCTGGGTGAAATAGCGACCCTTGGCTGAGACTCAGCGGCAAATTGGAGGGACGCATGGTGTAAATAGGGTGCAACATTAACTTCGTATATGAGTTCGCTAAGATACTCATCAAGCAGACTGCTGACGACGGGTCGACCTACACGGAAGACATGAGGCGCTTACAAGTACg                                                                                             >  1:45072/1‑301 (MQ=255)`  
                                                                                                                                                                                                             `tctgggttcggagatgtgtataagagacagctctATTCATATTCCAACAATTTATGGTGCAAACTTCGGGAGGGGCAAAATCACTGTATTGGATCCTGATGGTAAGATAACCGTGTTTGAGCAACCTACGGCTGGGTGGAATAGCGACCCTTGGCTGAGACTCAGCGGTAACTTGGAGGGACGCATGGTGTACATTGGGTTCAACATTAACTTCGTATATGAGTTCTCTAAGTTCCTCATCAAGCAGACTGCCGACGACGGGTCTACCTCCACGGAAGACATTGGGCGCTTACAGTTACGc                                                                                            <  1:81990/271‑1 (MQ=255)`  
                                                                                                                                                                                                             `tGGAACATACAACGATGACACATTCACTACCGCTATTCATATTCCAACAATTTATGGTGCAAACTTCGGTAGGGGCAAAATCACTGTATTGGATCCTGATGGTAAGATAACCGTGTTTGAGCAACCTACGGCTGGGTGGAATAGCGACCCTTGGCTGAGACTCAGCGGTAACTTGGAGGGACGCATGGTGTACATTGGGTTCAACATTAACTTCGTATATGAGTTCTCTAAGTTCCTCATCAAGCAGACTGCCGACGACGGGTCTACCTCCACGGAAGACATTTGGCGCTTACAGTTACGc                                                                                            <  1:111664/301‑1 (MQ=255)`  
                                                                                                                                                                                                             `gggAACATACAACGATGACACATTCACTACCTCTATTCATATTCCAACAATTTATGGTGCAAACTTCGGGAGGGGCAAAATCACTGTATTGGATCCTGATGGTAAGATAACCGTGTTTGAGCAACCTACGGCTGGGTGGAATAGCGACCCTTGGCTGAGACTCAGCGGTAACTTGGAGTGACGCATGGTGTACATTGGGTTCAACATTAACTTCGTATATGAGTTCTCTAAGTTCCTCATCAAGCAGACTGCCGACGACGGGTCTACCTCCACGGAAGACATTGGGCGCTTACAGTTACGc                                                                                            <  1:68824/300‑1 (MQ=255)`  
                                                                                                                                                                                                               `gAACATACAACGATGACACATTCACTACCTCTATTCATATTCCAACAATTTATGGTGCAAACTTCGGGAGGGGCAAAATCACTGTATTGGATCCTGATGGTAAGATAACCGTGTTTGAGCAACCTACGGCTGGGTGGAATAGCGACCCTTGGCTGAGACTCAGCGGTACCTTGGAGGGACGCATGGTGTACATTGGGTTCAACATTAACTTCGTATATGAGTTCTCTATGTTCCTCATCAAGCAGACTGCCGACGACGGGCCTACCTCCCCGGAAGACATTGGGCGCTTACAGTTACGccc                                                                                          >  1:275776/1‑300 (MQ=255)`  
                                                                                                                                                                                                                              `acacATTCACTACCTCTATTCATATTCCAACAATTTATGGTGCAAACTTCGGGAGGGGCAAAATCACTGTATTGGATCCTGATGGTAAGATAACCGTGTTTGAGCAACCTACGGCTGGGTGGAATAGCGACCCTTGGCTGAGACTCAGCGGTAACTTGGAGGGACGCATGGTGTACATTGGGTTCAACATTAACTTCGTATATGAGTTCTCTAAGTTCCTCATCAAGCAGACTGCCGACGACGGGTCTACCTCCACGGAAGACATTGGGCGCTTACAGTTACGCCGAGCGTGGGTTAACTa                                                                           <  1:164433/301‑1 (MQ=255)`  
                                                                                                                                                                                                                                  `gagacagaCCTCTATTCATATTCCAACAATTTATGGTTCAAACTTCGGTAGGGGCAAAATCACTGTATTGGATCCTGATGGTAAGATAACCGTGGTTGAGCAACCTACGGCTGGGTGGAATAGCGACCCTTGGCTGAGACTCAGCGGTAACTTGGAGGGACGCATGGTGTACATTGGGTTCAACATTAACTTCGTATATGAGTTCTCTAAGTTCCTCATCAAGCAGACTGCCGACGACGGGTCTACCTCCACGGAAGACATTGGGCGCTTACAGTTACGCCGAGCGTGGGTTAACTACgag                                                                       <  1:283209/294‑1 (MQ=255)`  
                                                                                                                                                                                                                                  `aTTCACTACCTCTATTCATATTCCAACAATTTATGGTGCAAACTTCGGGAGGGGCAAAATCACTGTATTGGATCCTGCTGGTAAGATAACCGTGTTTGAGCAACCTACGGCTGGGTGGAATAGCGACACTTGGCTGAAACTCAGCGGTAACTTGGAGGGACGCATGGTGTACATTGGGTTCAACATTAACTTCGTATATGAGTTCTCTAAGTTCCTCATCAAGCAGACTGCCGACGACGGGTCTACCTCCACGGCAGACATTGGGCGCTTACAGTTACGCCGAGCGTGGGTTAACTACgag                                                                       >  1:137869/1‑301 (MQ=255)`  
                                                                                                                                                                                                                                   `ttCACTACCTCTATTCATATTCCAACAATTTATGGTGCAAACTTCGGGAGGGGCAAAATCACTGTATTGGATCCTGATGGTAAGATAACCGTGTTTGAGCAACCTACGGCTGGGTGGAATAGCGACCCTTGGCTGAGACTCAGCGGTAACTTGGAGGGACGCATGGTGTACATTGGGTTCAACATTAACTTCGTATATGAGTTCTCTAAGTTCCTCATCAAGCAGACTGCCGACGACGGGTCTACCTCCACGGAAGACATTGGGCGCTTACAGTTACGCCAAGCgctgtctcttatacaca                                                                      >  1:174970/1‑285 (MQ=255)`  
                                                                                                                                                                                                                                   `ttCACTACCTCTATTCATATTCCAACAATTTATGGTGCAAACTTCGGGAGGGGCAAAATCACTGTATTGGATCCCGATGGTAAGATAACCGTGTTTGAGCAACCTACGGCTGGGTGGAATAGCGACCCTTGGCTGAGACTCAGCGGTAACTTGGAGGGACGCATGGTGTACATTGGGTTCAACATTAACTTCGTATATGAGTTCTCTAAGTTCCTCATCAAGCAGACTGCCGACGACGGGTCTACCTCCACGGAAGACATTGGGCGCTTACAGTTACGCCGAGCGTGGGTTAACTACgaga                                                                      >  1:124356/1‑301 (MQ=255)`  
                                                                                                                                                                                                                                   `ttCAATACCTCTACTCATATTACACCAAGTTATGGTGCAAACTTCGGGAGGGGCAAAATCACTGTATTGGATCCTGATGGTAAGATAACCGTGTTTGAGCAACCTACGGATGGGTGGAATAGCGACCCTTGGCTGAGACTCAGCGGTAACTTGGAGTGACGCATGGTGTACATTGGGTTCAACATTAACTTCGTATATGAGTTCTCTAAGTTCCTCATCAAGCAGACTGCCGACGACGGGTCTACCTACACGGAAGACATTGGGCGCTTACAGTTACGCCGAGCGTGGGTTAACTACgaga                                                                      <  1:177888/301‑1 (MQ=255)`  
                                                                                                                                                                                                                                       `cTACCTCTATTCATATTCCAACAATTTATGGTGCAAACTTCGGGAGGGGCAAAATCACTGTATTGGATCCTGATGGTAAGATAACCGTGTTTGAGCAACCTACGGCTGGGTGGAATAGCGACCCTTGGCTGAGACTCAGCGGTAACTTGGAGGGACGCATGGTGTACATGGGGTTCAACATTAACTTCGTATATGAGTTCTCTAAGTTCCCCATCAAGCAGACTGCCGACGACGGGTCTACCTCCACGGAAGACATTGGGCGCTTACAGTTACGCCGAGCGTGGGTTAACTACGAGAActc                                                                  >  1:258949/1‑301 (MQ=255)`  
                                                                                                                                                                                                                                          `ccTCTATTCATATTCCAACAATTTATGGTGCAAACTTC‑GGAAGGGCAAAATCACTGTATTGGATCCTGATGGTAAGATAACCGTGTTTGAGCAACCTACGGCTGGGTGGAATAGCGACCCTTGGCTGAGACTCAGCGGTAACTTGGAGGGACGCATGGTGTACATTGGGTTCAACATTAACTTCGTATATGAGTTCTCTAAGTTCCTCATCAAGCAGACTGCCGACGACGGGTCTACCTCCACGGAAGACATTGGGCGCTTACAGTTACGCCGAGCGTGGGTTAACTACGAGAACTCTGGt                                                              >  1:228006/1‑301 (MQ=255)`  
                                                                                                                                                                                                                                           `ctctATTCCTATTCCAACAATTTATGGTGCAAACTTCGGGAGGGGCAAAATCACTGTATTGGATCCTGATGGTAAGATAACCGTGTTTGAGCAACCTACGGCTGGGTGGAATAGCGACCCTTGGCTGAGACTCATCGGTAACTTGGAGGGACGCATGGTGTACATTGGGTTCAACATTAACTTCGTATATGAGTTCTCTAAGTTCCTCATCAAGCAGACTGCCGACTACGGGTCTACCTCCACGGAAGACATTGGGCGCTTACAGTTACGCCGAGCGTgggctgtctcttatacacatctc                                                              >  1:290125/1‑281 (MQ=255)`  
                                                                                                                                                                                                                                             `ctATTCATATTCCAACAATTTATGGTGCAAACTTCGGGAGGGGCAAAATCACTGTATTGGATCCTGATGGTAAGATAACCGTGTTTGAGCAACCTACGGCTGGGTGGAATAGCGACCCTTGGCTGAGACTCAGCGGTAACTTGGAGGGACGCATGGTGTACATTGGGTTCAACATTAACTTCGTATATGAGTTCTCTAAGTTCCTCATCAAGCAGACTGCCGACGACGGGTCTACCTCCACGGCAGACATTGGGCGCTTACAGTTACGCCGAGCGTGGGTTAACTACGAGAACTCTGGTAc                                                            >  1:77667/1‑301 (MQ=255)`  
                                                                                                                                                                                                                                               `aTTCATATTCCAACAATTTATGGTGCAAACTTCGGGAGGGGCAAAATCACTGTATTGGATCCTGATGGTAAGATAACCGTGTTTGAGCAACCTACGGCTGGGTGGAATAGCGACCCTTGGCTGAGACTCAGCGGTAACTTGGAGGGACGCATGGTGTACTTTGGGTTCAACATTAACTTCGTATATGAGTTCTCTAAGTTCCTCATCAAGCGGACTGCCGACGACGGGTCTACCTCCACGGAAGACATTGGGCGCTTACAGTTACGCCGAGCGTGGGTTAACTACgagctgtctcttatac                                                          >  1:141792/1‑288 (MQ=255)`  
                                                                                                                                                                                                                                                      `ttCCAACAATTTATGGTGCAAACTTCGGGAGGGGCAAAATCACTGTATTGGATCCTGATGGTAAGATAACCGTGTTTGAGCAACCTACGGCTGGGTGGAATAGCGACCCTTGGCTGAGACTCAGCGGTAACTTGGAGGGACGCATGGTGTACATTGGGTTCAACCTTAACTTCGTATATGAGTTCTCTAAGTTCCTCATCAAGCAGACTGCCGACGACGGGTCTACCTCCACGGACGACATTGGGCGCTTACAGTTACGCCGATCGTGGGTTAACTACGAGAACTCTGGTACTTTTGCCAt                                                   >  1:335570/1‑301 (MQ=255)`  
                                                                                                                                                                                                                                                      `ttCCAACAATTTATGGTGCAAACTTCGGGAGGGGCAAAATCACTGTATTGGATCCTGATGGTAAGATAACCGTGTTTGAGCAACCTACGGCTGGGTGGAATAGCGACCCTTGGCTGAGACTCAGCGGTAACTTGGAGGGACGCATGGTGTACATTGGGTTCAACATTAACTTCGTATATGAGTTCTCTAAGTTCCTCATCAAGCAGACTGCCGACGACGGGTCTACCTCCACGGAAGACATTGGGCGCTTACAGTTACGCCGAGCGTGGGTTAACTACGAGAACTCTGGTACGTTTGACAt                                                   >  1:213395/1‑301 (MQ=255)`  
                                                                                                                                                                                                                                                           `acaaTTTATGGTGCAAACTTCGGGAGGGGCAAAATCACTGTATTGGATCCTGATGGTAAGATAACCGTGTTTGAGCAACCTACGGCTGGGTGGAATAGCGACCCTTGGCTGAGACTCAGCGGTAACTTGGAGGGACGCATGGTGTACATTGGGTTCAACATTAACTTCGTATATGAGTTCTCTAAGTTCCTCATCAAGCAGACTGCCGACGACGGGTCTACCTACACGGAAGACATTGGGCGCTTACAGTTACGCCGAGCGTGGGTTAACTACgagactgtctcttatacacatctccgag                                              >  1:308523/1‑277 (MQ=255)`  
                                                                                                                                                                                                                                                            `caaTTTATGGTGCAAACTTCGGGAGGGGCAAAATCACTGTATTGGATCCTGATGGTAAGATAACCGTGTTTGAGCAACCTACGGCTGGGTGGAATAGCTACCCTTGGCTGAGACTCAGCGGTAACTTGGAGGGACGCATGGTGTACATTGTGTTCAACATTAACTTCGTATATGAGTTCTCTAAGTTCCTCATCAAGCAGACTGCCGACGACGGGTCTACCTCCACGGAAGACATTGGGCGCTTACAGTTACGCCGAGCGTGGGTTAACTACGAGAACTCTGGTACGTTTGACATTTATGt                                             >  1:121341/1‑301 (MQ=255)`  
                                                                                                                                                                                                                                                                  `aTGGTGCACACTTCGGGAGGTGCAAAATCACTGTATTGGATCCTGATGGTAAGATAACCGTGTTTGAGCAACCTACGGCTGGGTTGAATAGCGACCCTTGGCTGAGACTCAGCGGTAACTTGGAGGGACGCATGGTGTACATTGGGTTCAACATTAACTTCGTATATGAGTTCTCTAAGTTCCTCATCAGGCAGACTGCCGACGACGGGTCTACCTCCACGGAAGACATTGGGCGCTTACAGTTACGCCGAGCGTGGGTTAACTACGAGAACTCTGGTACGTTTGACATTTATGTTGAGaa                                       >  1:59224/1‑301 (MQ=255)`  
                                                                                                                                                                                                                                                                          `aaCTTTGGGAGGGGCAAAATCACTGTATTGGATCCTGATGGTAAGATAACCGTGTTTGAGCAACCTACGGCTGGGTGGAATAGCGACCCTTGGCTGAGACTCAGCGGTAACTTGGAGGAACGCATGGTGTACATTGGGTTAAACATTAACTTCGTATATGAGTTCTCTAAGTTCCTCATCAAGCAGACTGCCGACGACGGGTCTACCTCCACGGAAGACATTGGGCGCTTACAGTTACGCCGAGCGTGGGTTAACTACGAGAACTCTGGTACGTTTGACATTTATGTTGAGAACCAATCGt                               >  1:192708/1‑301 (MQ=255)`  
                                                                                                                                                                                                                                                                                          `aaaTCACTGTATTGGATCCTGATGGTAAGATAACCGTGTTTGAGCAACCTACGGCTGGGTGGAATAGCGACCCTTGGCTGAGACTCAGCGGTAACTTGGAGGGACGCATGGTGTACATTGGGTTCAACATTAACTTCGTATATGAGTTCTCTAAGTTCCTCATCAAGCAGACTGCCGACGACGGGTCTACCTCCACGGAAGACATTGGGCGCTTACAGTTACGCCGAGCGTGGGTTAACTACGAGAACTCTGGTACGTTTGACATTTATGTTGAGAACCAATCGTCTAACTGGAAGTacac               <  1:338011/301‑1 (MQ=255)`  
                                                                                                                                                                                                                                                                                          `aaaTCACTGGATTGGATCCTGATGGTAAGGTAACCGTGTTTGAGCAACCTACGGCTGGGTGGAATAGCGACCCTTGGCTGAGACGCAGCGGTAACTTGGAGGGACGCATGGTGTACATTGGGTTCAACATTAACTTCGTATATGAGTTCTCTAAGTTCCTCATCAAGCAGACTGCCGACGACGGGTCTACCTCCACGGAAGACATTGGGCGCTTACAGTTACGCCGAGCGTGGGTTAACTACGAGAACTCTGGTACGTTTGACATTTATGTTGAGAACCAATCGTCTAACTGGAAGTacac               <  1:230322/301‑1 (MQ=255)`  
                                                                                                                                                                                                                                                                                             `tCACTGTATTGGATCCTGATGGTAAGATAACCGTGTTTGAGCAACCTACGGCTTGGTGGAATAGCGACCCTTGGCTGAGACTCAGCGGTAACTTGGAGGGACGCATGGTGTACATTGGGTTCAACATTAACTTCGTATATGAGTTCTCTAAGTTCCTCATCAAGCAGACTGCCGACGA‑GGGTCTACCTCCACGGAAGCCATTGGGCGCTTACAGTTACGCCGAGCGTGGGTTAACTACGAGAACTCTGGTACGTTTGACATTTATGTTGAGAACCCATCGTCTAACTGGAAGTACACAATg           >  1:126911/1‑301 (MQ=255)`  
                                                                                                                                                                                                                                                                                             `tCACTGTATTGGATCCTGATGGTAAGATAACCGTGTTTGAGCAACCTACGGCTGGGTGGAATAGCGACCCTTGGCTGAGACTCAGCGGTAACTTGGAGGGACGCATGGTGTACATTGGGTTCAACATTAACTTCGTATATGAATTCTCTAAGTTCCTCATCAAGCAGACTGCCGACGACGGGTCTACCTCCACGGAAGACATTGGGCGCTTACAGTTACGCCGAGCGTGGGTTAACTATGAGAACTCTGGTACGTTTGACATTTATGTTGAGAACCAATCGTCTAACTGGAAGTACACAAt            >  1:211812/1‑301 (MQ=255)`  
                                                                                                                                                                                                                                                                                             `gagacagaTTGGATCCTGATGGTAAGATAACCGTGTTTGAGCAACCTACGGCTGGGTGGAATAGCGACCCTTGGCTGAAACTCAGCGGTAACTTGGAGGGACGCATGGTGTACATTGGGTTCAACATTAACTTCGTATATGAGTTCTCTAAGTTCCTCATCAAGCAGACTGCCGACGACGGGTCTACCTCCACGGAAGACATTGGGCGCTTACAGTTACGCCGAGCGTGGGTTAACTACGAGAACTCTGGTACGTTTGACATTTATGTTGAGAACCAATCGTCTAACTGGAAGTACACAAt            <  1:219041/294‑1 (MQ=255)`  
                                                                                                                                                                                                                                                                                                `cTGTATTGGATCCTGATGGTAAGATAACCGTGTTTGAGCAACCTACGGCTGGGTGGAATAGCGACCCTTGGCTGAGACTCAGCGGTAACTTGGAGGGACGCATGGTGTACATTGGGTTCAACATTAACTTCGTATATGAGTTCTCTAAGTTCCTCATCAAGCAGACTGCCGACGACGGGTCTACCTCCACGGAAGACATTGGGCGCTTACAGTTACGCCGAGCGTGGGTTAACTACGAGAACTCTGGTACGTTTGACATTTATGTTGAGAACCAATCGTCTAACTGGAAGTACACAATGGc         >  1:7507/1‑301 (MQ=255)`  
                                                                                                                                                                                                                                                                                                  `gTATTGGATCCTGATGGTAAGATAATCGTGTTTGAGCAACCTACGGCTGGGTGGAATAGCGACCCTTGGCTGAGACTCAGCGGTAACTTGGAGGTACGCATGGTGTACATTGGGTTCAACATTAACTTCGTATATGAGTTCTCTAAGTTCCTCATCAAGCAGACTGCCGACGACGGGTCTACCTCCACGGAAGACATTGGGCGCTTACAGTTACGCCGAGCGTGGGTTAACTACGAGAACTCTGGTACGTTTGACATTTATGTTGAGAACCAATCGTCTAACTGGAAGTACACAATGGCTg       >  1:198463/1‑301 (MQ=255)`  
                                                                                                                                                                                                                                                                                                  `gTATTGGATCCTGATGGTAAGATAACCGTGTTTGAGCAACCTACGGCTGGGTGTAATAGCGACCCTTGGCTGAGACTCAGCGGTAACTTGGATGGACGCATGGTGTACATTGGGTTCAACATTAACTTCGTATATGAGTTCTCTAAGTTCCTCATCAAGCAGACTGCCGACGACGGGTCTACCTCCACGGAAGACATTGGGCGCTTACAGTTACGCCGAGCGTGGGTTAACTACGAGAACTCTGGTACGTTTGACATTTATGTTGAGAACTAATCGTCTACATGGCAGTAATCAATGGCTg       >  1:206967/1‑301 (MQ=255)`  
                                                                                                                                                                                                                                                                                                  `gTATTGGATCCTGATGGTAAGATAACCGTGTTTGAGCAACCTACGGCTGGGTGGAATAGCGACCCTTGGCTGAGACTCAGCGGTAACTTGGAGGGACGCATGGTTTACATTGGGTTCAACATTAACTTCGTATATGAGTTCTCTAAGTTCCTCATCAAGCAGACTGCCGACGACGGGTCTACCTCCACGTAAGACATTGGGCGCTTACAGTTACGCCGAGCGTGTGTTCACTACGAGAACTCTGGTACGTTTGACATTTATGTTGAGAACCAATCGTCTAACTGGAAGTACACAATGGcct       >  1:108243/1‑299 (MQ=255)`  
                                                                                                                                                                                                                                                                                                    `aTTGGATCCTGATGGTAAGATAACCGTGTTTGAGCAACCTACGGCTGGGTGGAATAGCGACCCTTGGCTGAGACTCAGCGGTAACTTGGAGGGACGCATGGTGTACATTGGGTTCAACATTAACTTCGTATATGAGTTCTCTAAGTTCCTCATCAAGCAGACTGCTGACGACGGGTCTACCTCCACGGAAGACATTGGGCGCTTACAGTTACGCCGAGCGTGGGTTAACTACGAGAACTCTGGTACGTTTGACATTTATGTTGAGAACCAATCGTCTAACTGGAAGTACACAATGGCTGGt     >  1:251282/1‑301 (MQ=255)`  
                                                                                                                                                                                                                                                                                                    `aTTGGATCCTGATGGTAAGATAACCGTGTTTGAGCAACCTACGGCTGGGTGGAATAGCGACCCTTGGCTGAGACTCAGCGGTAACTTGGAGGGACGCATGGTGTACATTGGGTTCAACATTAACTTCGTATATGAGTTCTCTAAGTTCCTCATCAAGCAGACTGCCGACGACGGGTCTACCTCCACGGAAGACATTGGGCGCTTACAGTTACGCCGAGCGTGGGTTAACTACGAGAACTCTGGTACGTTTGACATTTATGTTGAGAACCAATCGTCTAACTGGAAGTACACAATGGATGGt     >  1:158370/1‑301 (MQ=255)`  

                                                                                                                                                                                                                                                                                                          `|`                                                                                                                                                                                                                                                                                                           
`GGCAACAGTCGTGGTCTCATTGGGACTTTGGGGAAAACGTACAGGTTCTAGCTTGTCAGAGTATCAGCTCAGATATGTATGTGATTCTTCGCAATGAGTTCAATACGTTCCTAGCTAGAATCTCTTTCACTAAGAACGCCATTGACTTACAGGGAGAACCCTATCGTGCCTTTATGGACATGAAGATTCGATACACGATTCCTAGTGGAACATACAACGATGACACATTCACTACCTCTATTCATATTCCAACAATTTATGGTGCAAACTTCGGGAGGGGCAAAATCACTGTATTGGAGCCTGATGGTAAGATAACCGTGTTTGAGCAACCTACGGCTGGGTGGAATAGCGACCCTTGGCTGAGACTCAGCGGTAACTTGGAGGGACGCATGGTGTACATTGGGTTCAACATTAACTTCGTATATGAGTTCTCTAAGTTCCTCATCAAGCAGACTGCCGACGACGGGTCTACCTCCACGGAAGACATTGGGCGCTTACAGTTACGCCGAGCGTGGGTTAACTACGAGAACTCTGGTACGTTTGACATTTATGTTGAGAACCAATCGTCTAACTGGAAGTACACAATGGCTGGTGCC  >  NC_001604.1/26448‑27043`

  

Alignment Legend

Aligned base mismatch/match (shaded by quality score): `ATCG/ATCG` `< 3 ≤` `ATCG/ATCG` `< 11 ≤` `ATCG/ATCG` `< 24 ≤` `ATCG/ATCG` `< 36 ≤` `ATCG/ATCG` `< 38 ≤` `ATCG/ATCG`

Unaligned base: `atcg`    Masked matching base: `atcg`    Alignment gap: `‑`    Deleted base: `‑`
